# Acetabular Bone Preservation in H1 Ceramic Hip Resurfacing: A Comparison With A Conventional Metal‐on‐Metal Hip Resurfacing

**DOI:** 10.1002/jor.70221

**Published:** 2026-05-07

**Authors:** Yuki Yamamuro, Simon Harris, Rima Nasser, Amir Ardakani, Justin Cobb

**Affiliations:** ^1^ MSk Lab Imperial College London London UK; ^2^ Department of Orthopaedic Surgery Kanazawa University Kanazawa Japan

**Keywords:** acetabular bone resection, cementless ceramic‐on‐ceramic hip resurfacing, ceramic‐on‐ceramic hip resurfacing, hip resurfacing, metal‐on‐metal hip resurfacing

## Abstract

This study compared the volume of acetabular bone resection between an anatomically designed cementless ceramic‐on‐ceramic hip resurfacing arthroplasty (H1HR) and metal‐on‐metal hip resurfacing (Birmingham Hip Resurfacing; BHR). We hypothesized that the anatomical design of the H1 cup would reduce the need for medialization to avoid rim overhang, thereby preserving acetabular bone stock. A retrospective three‐dimensional simulation study was performed in 12 hips with osteoarthritis and femoral head size ≥ 48 mm. H1HR and BHR were virtually implanted using 3D planning software to minimize the risk of groin pain. The primary outcome was the volume of acetabular bone resection. Secondary outcomes included the position of the cup relative to the native hip centre and the cup non‐coverage ratio. H1 cups required a mean volume of acetabular bone resection of 11 cm^3^, while BHR cups required 17 cm^3^. This 35% reduction was statistically significant. H1 cups had less medialization (−1 mm vs. −6 mm), superior displacement (−1 mm vs. −2 mm), and posterior displacement (−1 mm vs. −3 mm). The mean cup non‐coverage ratio was not significantly different (10% vs. 8%). The anatomically designed H1HR achieved a significant 35% reduction in acetabular bone resection compared with BHR. This substantial volume of bone preservation was primarily attributed to the design enabling cup placement with minimal medialization, without increasing the cup non‐coverage ratio. Although further validation in cadaveric or postoperative imaging studies is needed, H1 may be a promising option for bone preservation, particularly for young, active patients who might require future revision surgery.

## 1 Introduction

Hip resurfacing arthroplasty (HRA) is an alternative to total hip arthroplasty (THA) for end‐stage hip osteoarthritis. Potential advantages of HRA include reduced risk of dislocation due to the larger femoral head, greater resistance to wear, improved joint function, restoration of physiological gait, and the potential to return to a wide range of sporting activities [1, 2, 3]. In addition, HRA offers the advantage of femoral bone stock preservation, which is beneficial for potential future revisions [1, 4, 5]. However, previous studies have raised concerns about acetabular bone resection [5, 6, 7, 8, 9, 10, 11, 12]. The H1 hip resurfacing (H1HR) was developed as a cementless, ceramic‐on‐ceramic (CoC) HRA. CoC HRA has emerged as a promising alternative that may overcome the limitations of metal‐on‐metal (MoM) designs [13]. Early results suggest that CoC HRA could be applied to a broader range of patients, including women and those with smaller femoral heads, due to its improved biocompatibility, reduced wear, and absence of metal ion‐related complications [14]. The H1 cup features an anatomically contoured, hemispherical acetabular cup with a specially designed rim to reduce iliopsoas impingement (IPI) and soft tissue irritation [15]. This design feature reduces the surface area of the cup by approximately 9% compared with a cup of the same nominal size without the cutout, regardless of cup size. While reducing the risk of soft tissue impingement, in dysplastic hips the rim must be strong enough to withstand load with a substantial area of unsupported device. For this reason, the wall thickness of the H1 cup was increased from 3 to 3.5 mm. This increase in wall thickness would theoretically lead to greater acetabular bone resection. Nevertheless, the impact of the H1 cup design and increased thickness on acetabular bone stock preservation remains unclear.

We hypothesized that the anatomical design of the H1 cup, despite having a 0.5 mm thicker wall, may facilitate more lateralized cup positioning, which could help preserve acetabular bone stock compared with a widely used MoM HRA (Birmingham Hip Resurfacing; BHR) [16]. The aim of this study was to compare the volume of acetabular bone resection between H1HR and BHR.

## 2 Materials and Methods

### 2.1 Study Design and Patient Participants

This retrospective three‐dimensional (3D) simulation study used a 3D templating software (3D Hip Planner designed by Simon Harris), which calculates the volume of acetabular bone resection based on virtual implant placement. This study included 12 consecutive hips (9 males and 3 females) with hip osteoarthritis who underwent HRA and had a femoral head size of ≥ 48 mm by a single surgeon (Justin Cobb) at a single institution. The use of computed tomography (CT) images was approved by the patients and the institutional ethics committee. The mean age was 56 ± 11 years (range: 34–70 years). Seven hips were templated on the left side and five on the right. Six of the hips were dysplastic, as defined by the criteria of a lateral centre‐edge (CE) angle of < 25° [17].

The overall mean CE angle was 25 ± 6°, with a mean of 30 ± 2° in non‐dysplastic hips and 20 ± 3° in dysplastic hips.

### 2.2 HRA Simulation

The pelvic position was standardized with reference to the anterior pelvic plane [18]. The coordinate system of the femur was defined on the femoral posterior condylar plane [19]. Simulations of H1HR and BHR were performed as previously described [20]. During the HRA simulation, if the final cup size and position were not acceptable, the femoral head component was revisited, and its size was readjusted accordingly.

### 2.3 Femoral Head Component

**Figure 1:**
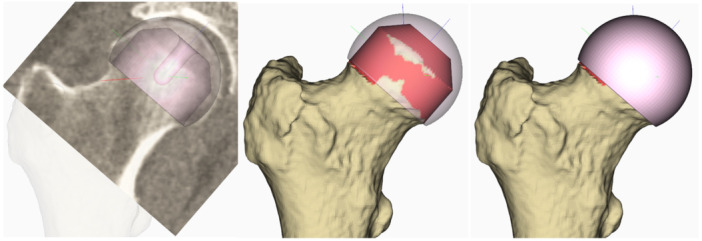
Femoral head placement. Implant positioning was adjusted to avoid notching, provide cortical support, maintain valgus stem orientation, and avoid leg length shortening.

The femoral head centre was determined by fitting a sphere to the native femoral head surface. The size, angle, and position were adjusted to meet the following conditions: (a). Avoid notching of the femoral neck; (b). Achieve cortical overlap at both the medial and lateral head‐neck junctions; (c). Maintain a valgus stem orientation relative to the native neck axis, typically around 140°, with adjustments made in cases of coxa vara or valga; (d). Provide adequate lateral bone support; (e). Align the medial rim of the implant at the medial head‐neck junction; (f). Avoid leg length shortening (Figure 1).

### 2.4 Cup Component

The corresponding cup size was selected based on the femoral head size. For H1HR, the cup was 7 mm larger than the H1 head, whereas for BHR, the cup was 6 mm larger than the BHR head. The target angle was 45° inclination and 20° anteversion for the H1 cup, and 40° and 20° for the BHR cup, based on the respective surgical technique manuals.

**Figure 2:**
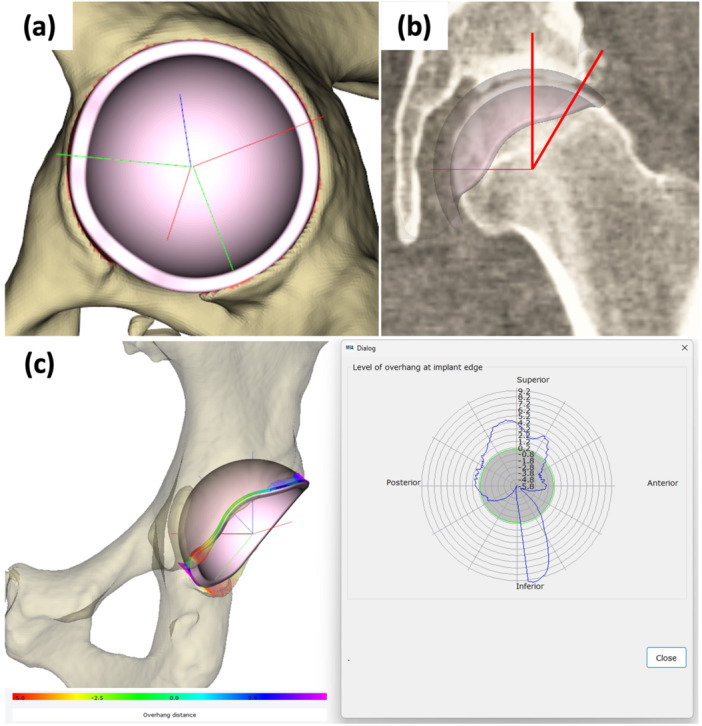
Cup placement. (a) Optimal positioning of the cup. To achieve an optimal fit, the cup is placed between the anterior and posterior bone columns, with its inferior edge set at the level of the distal part of the cotyloid notch. (b) The angle shown in red represents the cup‐CE angle, which was required to be greater than 8°. (c) Rim‐matching analysis to quantify cup overhang and embedding. The polar plot visualizes the measured distance (blue line) between the implant rim and the native acetabular rim (grey circle representing perfect alignment, with the native rim shown as a green outline). Positive values indicate overhang, and negative values indicate embedding. The interface is colour‐coded to represent the displacement magnitude, ranging from red (0 mm) to purple (5 mm). (CE, centre‐edge).

The cup position was adjusted to meet the following conditions: (a). Optimal cup fit with the acetabular bone was achieved by positioning the cup within the true acetabulum [21] (Figure 2a); (b). To ensure appropriate acetabular coverage and contact, defined as a cup‐CE angle greater than 8°, the vertical centre of the cup was allowed to shift superiorly by up to 3 mm [22, 23] (Figure 2b); (c). Avoid breaching the cortex of the acetabular fossa [24]; (d). Minimize the risk of IPI by avoiding anterior overhang, particularly in the anterosuperior region. If full containment was not feasible, anterior overhang was limited to less than 5 mm to prevent symptomatic IPI [25]. Regarding criterion (d), anterior overhang was evaluated with a rim‐matching analysis by measuring the circumferential distance between the implant and native rims. The degree of overhang or embedding was visualized in a polar plot (Figure 2c).

### 2.5 Acetabular Bone Resection Evaluation

**Figure 3:**
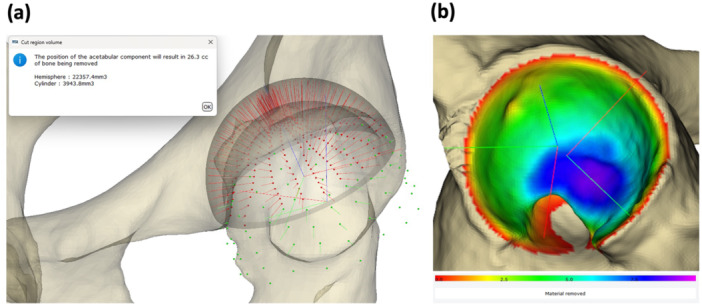
Evaluation of acetabular bone resection. (a) The volume of acetabular bone resection was calculated by summing the bone intersecting a virtual hemisphere, aligned with the final cup, and a cylinder along the reaming trajectory. (b) The colour map visualizes the depth of bone resection from the original acetabular surface. The colour scale indicates increasing depth from red (0 mm) to purple (10 mm), with depths exceeding 10 mm shown in black.

To evaluate acetabular bone resection, we calculated the total volume of acetabular bone resection (cm^3^) by modelling the reamed cavity with a corresponding hemisphere and cylinder. Additionally, the resection depth from the native acetabular surface was visualized using a colour map (Figure 3).

### 2.6 Cup Position Evaluation

The native hip centre was defined as the geometric centre of a best‐fit sphere to the surface landmarks of the acetabulum. Horizontal distance was defined as the mediolateral deviation from the native hip centre to the centre of the cup (positive = lateralization). Vertical distance was defined as the superoinferior deviation from the native hip centre to the centre of the cup (positive = inferiorization). Anterior–posterior distance was defined as the anteroposterior deviation from the native hip centre to the centre of the cup (positive = anteriorization).

### 2.7 Cup Non‐Coverage Evaluation

Cup non‐coverage ratio (%) was evaluated as the proportion of the implant surface not covered by host bone. The primary analysis was performed on the cup segment extending from the 8 o'clock to the 4 o'clock position, excluding the inferior fossa region, to emphasize the superior load‐bearing region and the anterior and posterior regions relevant to implant stability.

### 2.8 Primary and Secondary Outcomes

The primary outcome was the volume of acetabular bone resection. The secondary outcomes were the cup position relative to the native hip centre, assessed by measuring the horizontal, vertical, and anterior–posterior distances from the native hip centre to the centre of the cup, and the cup non‐coverage ratio.

### 2.9 Statistical Analyses

Analyses were performed using R version 4.4.2 (R Foundation for Statistical Computing, Vienna, Austria) [26]. The Shapiro–Wilk test was used to assess data for normality. For paired continuous variables with a normal distribution, the paired Student's *t*‐test was used. For non‐normally distributed data, the Wilcoxon signed‐rank test was applied. Data are presented as means with standard deviations (SDs) and 95% confidence intervals (CIs), or medians with interquartile ranges (IQRs). A two‐sided *p* < 0.05 was considered statistically significant.

The intra‐ and inter‐observer reliabilities of the measurements were assessed using the intraclass correlation coefficient (ICC). To evaluate intra‐observer reliability, one trained surgeon (Yuki Yamamuro) performed all measurements twice for each case with a 1‐month interval to minimize recall bias. To assess inter‐observer reliability, a second trained surgeon (Amir Ardakani) independently performed the same measurements. A two‐way random‐effects model with absolute agreement was used for the ICC analysis.

### 2.10 Sample Size Calculation

An a priori power analysis (G*Power software, version 3.1.9.7) for a two‐tailed paired *t*‐test (*α* = 0.05, power = 80%) indicated that a minimum of 5 pairs was required to detect a significant difference for the primary outcome. The parameters for this calculation were derived from a preliminary analysis (Supporting Information).

## 3 Results

### 3.1 Component Sizes Between H1HR and BHR

The median head size was 50 mm for the H1HR and 50 mm for the BHR. The median cup size was 57 mm for the H1HR and 56 mm for the BHR. There were no statistically significant differences in the median sizes of either the head (*p* = 0.62, Wilcoxon signed‐rank test) or the cup (*p* = 0.22, Wilcoxon signed‐rank test), although the cup size was numerically larger in the H1HR (Table 1).

**Table 1:** Head/cup size combinations for H1HR and BHR.

Variable	H1HR	BHR	*p*‐value
Median head size, mm (IQR)	50 (48–54)	50 (48–53)	0.62*
Median cup size, mm (IQR)	57 (55–61)	56 (54–59)	0.22*

*Note:* All values are presented as median (IQR).

Abbreviations: BHR, Birmingham hip resurfacing; H1HR, H1 hip resurfacing; IQR, interquartile range.

* Wilcoxon signed‐rank test.

Regarding the modal sizes, the most frequently used head size was 48 mm for both groups, while the modal cup size was 55 mm for the H1HR and 54 mm for the BHR.

**Figure 4:**
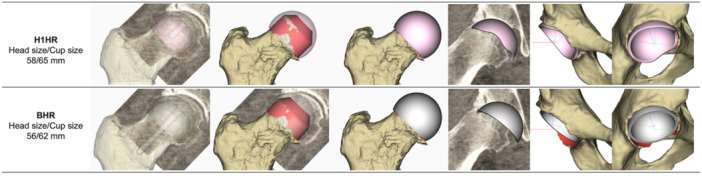
Representative case showing different implant sizes between H1HR (58/65 mm) and BHR (56/62 mm). (BHR, Birmingham hip resurfacing; H1HR, H1 hip resurfacing).

Different implant sizes were selected for 4 pairs (H1HR head/cup size vs. BHR head/cup size): 58/65 mm versus 56/62 mm, 54/61 mm versus 52/58 mm, 56/63 mm versus 54/60 mm, and 52/59 mm versus 50/57 mm (Figure 4). The detailed distribution of implant sizes is provided in Supporting Table SⅠ.

### 3.2 Primary Outcome

**Figure 5:**
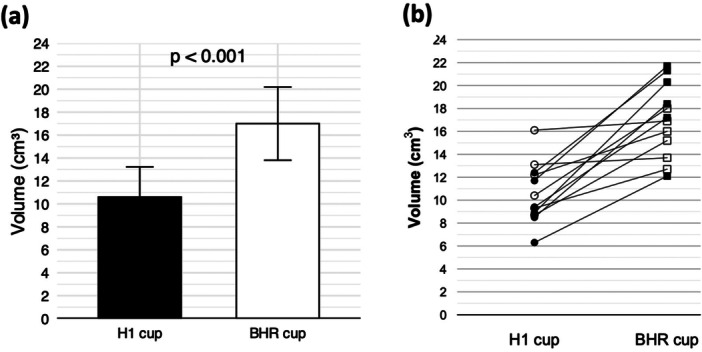
Comparison of Acetabular Bone Resection Volume between H1cup and BHR cup. (a) Mean bone resection volume was significantly lower with the H1 cup (black bar) compared with the BHR cup (white bar) (*p* < 0.001, paired Student's *t*‐test). Values are presented as means ± standard deviations. (b) The slopegraph demonstrates that the H1 cup resulted in less resection in all 12 hips. Open symbols (○ for H1 and □ for BHR) indicate dysplastic hips, whereas filled symbols (● for H1 and ■ for BHR) represent non‐dysplastic hips. Each line connects paired H1 and BHR bone resection volumes from the same hip, showing consistently lower resection volumes with the H1 cup. (BHR, Birmingham hip resurfacing).

**Figure 6:**
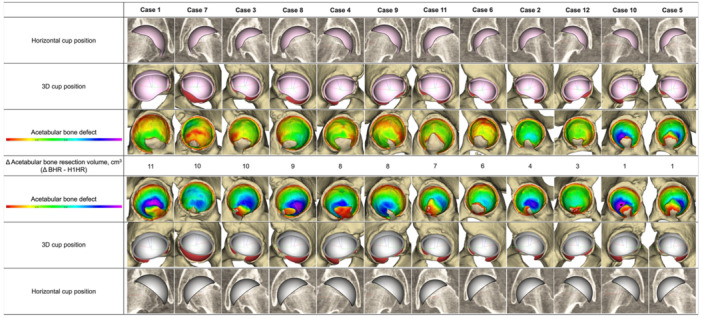
Cup position and acetabular bone resection volume between H1HR and BHR. Cases are ordered by descending Δ acetabular bone resection volume (Δ BHR–H1HR). For each hip, horizontal and 3D cup positions and bone defect colour maps are shown, with Δ volumes (cm^3^) indicated below. (3D, three‐dimensional; BHR, Birmingham hip resurfacing; H1HR, H1 hip resurfacing).

The volume of acetabular bone resection in the H1HR was 11 ± 3 cm^3^, compared with 17 ± 3 cm^3^ in the BHR. The volume of acetabular bone resection was significantly lower in the H1HR compared with the BHR (*p* < 0.001, paired Student's *t*‐test) (Table 2, Figure 5). The H1HR resulted in less acetabular bone resection than the BHR in all 12 hips, regardless of whether they were dysplastic hips or non‐dysplastic hips (Figure 5). The individual paired differences are illustrated in Figure 6.

**Table 2:** Comparison of acetabular bone resection volume, cup position, and cup non‐coverage ratio between H1HR and BHR.

Outcome measure	H1 cup	BHR cup	*p*‐value
Mean acetabular bone resection volume, cm^3^ (SD; 95% CI)	11 (3; 9–12)	17 (3; 15–19)	< 0.001*
Mean horizontal distance, mm (SD; 95% CI)	−1 (2; −2–0)	−6 (2; −7 to −4)	< 0.001*
Mean vertical distance, mm (SD; 95% CI)	−1 (1; −1–0)	−2 (1; −3 to −2)	< 0.001*
Mean anterior–posterior distance, mm (SD; 95% CI)	−1 (1; −2 to −1)	−3 (1; −4 to −2)	< 0.001*
Mean cup non‐coverage ratio, % (SD; 95% CI)	10 (4; 7–13)	8 (4; 6–10)	0.235*

*Note:* All values are presented as mean (SD; 95% CI).

Abbreviations: BHR, Birmingham hip resurfacing; CI, confidence interval; H1HR, H1 hip resurfacing; SD, standard deviation.

* Paired Student's *t*‐test.

### 3.3 Secondary Outcome

**Figure 7:**
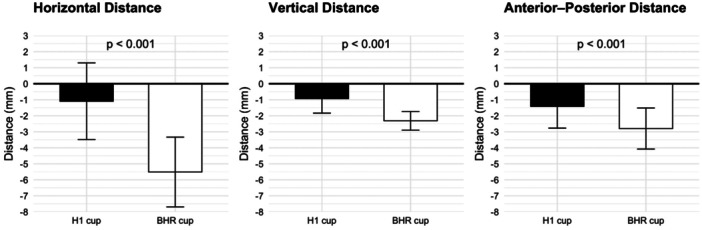
Comparison of cup position between H1 cup and BHR cup. Mean displacements of the H1 cup (black bars) and the BHR cup (white bars) are shown in three dimensions. All comparisons were performed using paired Student's *t*‐tests (*p* < 0.001). Values are presented as means ± standard deviations. (BHR, Birmingham hip resurfacing).

The mean horizontal distance was −1 ± 2 mm for the H1 cup compared with −6 ± 2 mm for the BHR cup, indicating significantly less medialization (*p* < 0.001, paired Student's *t*‐test). In the vertical direction, the mean displacement for the H1 cup was −1 ± 1 mm versus −2 ± 1 mm for the BHR cup, a statistically significant difference showing less superior displacement (*p* < 0.001, paired Student's *t*‐test). Similarly, the mean anterior‐posterior distance was −1 ± 1 mm for the H1 cup and −3 ± 1 mm for the BHR cup, which was also a significant difference (*p* < 0.001, paired Student's *t*‐test) (Table 2, Figure 7). The H1 cup was positioned significantly closer to the native hip centre in all three dimensions (Table 2, Figure 7). The mean cup non‐coverage ratio was 10 ± 4% for the H1 cup compared with 8 ± 4% for the BHR cup. The difference was not statistically significant (*p* = 0.235, paired Student's *t*‐test) (Table 2).

### 3.4 Reliability of Measurements

The ICCs for inter‐ and intra‐observer reliability were 0.87–0.98 and 0.94–0.99, respectively. Overall, the inter‐ and intra‐observer reliabilities were good to excellent for all parameters (Supporting Table SIⅠ).

## 4 Discussion

The principal finding of this study was that H1HR significantly reduced acetabular bone resection compared with the widely used BHR, achieving approximately 35% greater bone preservation. We demonstrated that this superior bone preservation is achieved through the H1 cup's anatomical design, which facilitates a more accurate 3D restoration of the native hip centre, specifically by markedly reducing medialization, without a significant increase in cup non‐coverage ratio.

One proposed advantage of HRA in younger patients is the preservation of bone to facilitate future revision surgery. HRA has been widely recognized for its superiority in preserving femoral bone and retaining the proximal femoral anatomy [4, 5, 9]. However, the preservation of acetabular bone remains a topic of debate [4, 5, 6, 7, 8, 9, 10, 11, 12, 27, 28, 29], particularly with the BHR, for which the evidence is inconclusive [5, 6, 7, 8, 9, 27, 28]. Some studies indirectly estimated bone resection via cup size or the cup/head ratio, which poorly reflects the actual volume resected. Only two studies have directly assessed acetabular bone resection [5, 7]. An experimental study using dry pelvic Sawbones by Crawford et al. reported a 311% increase in acetabular bone resection during BHR compared with THA (6 g vs. 2 g) [5]. Additionally, a prospective clinical study by Brennan et al. found a trend toward greater acetabular bone resection in BHR, although the difference was not statistically significant (14 g vs. 12 g) [7]. These studies have raised concerns about the ability of the BHR to preserve acetabular bone.

Therefore, this study aimed to evaluate whether the anatomically designed H1HR enables better preservation of acetabular bone compared with the BHR. Using 3D simulation with a standardized surgical protocol and anatomically matched datasets, we found that the volume of acetabular bone resection was significantly lower with the H1HR than with the BHR (11 cm^3^ vs. 17 cm^3^, *p* < 0.001). The clinical significance of this acetabular bone preservation is substantial. For young, active patients who are the primary candidates for HRA, preserving acetabular bone stock during the primary surgery provides greater flexibility in implant selection and facilitates a better reconstruction should future revision surgery be required. This study suggests that H1HR may offer a new advantage in acetabular bone preservation in addition to the established benefits of CoC bearings, such as excellent biocompatibility, low wear, and the avoidance of metal ion‐related complications.

Our analysis showed no statistically significant difference in the median head or cup sizes between the groups; however, larger cups tended to be used in H1HR. This tendency can be explained by a systematic difference in the internal diameter of the femoral heads between the two designs. Based on measurements from STL (Stereolithography) models for H1 and magnification‐corrected 2D templates for BHR (with a minor uncertainty from template line thickness), the BHR head internal diameters were consistently 1 to 2 mm larger than those of the H1 across nominal sizes from 48 mm to 58 mm. Because cup size selection is closely linked to femoral head size, this dimensional difference likely led to different nominal head choices, particularly in patients with larger femoral heads, and consequently influenced the final cup size.

Interestingly, despite the H1 cup having a median diameter that was more than 1 mm larger than the BHR cup (57 mm vs. 56 mm), the volume of acetabular bone resection was about 35% lower in the H1HR compared with the BHR. This apparent paradox may be reconciled by the accuracy of hip centre restoration, particularly the degree of medialization. Our secondary outcomes analysis revealed that the H1 cup restored the native hip centre more accurately than the BHR cup, with statistically significant differences in all three planes (horizontal, vertical, and anterior‐posterior). Notably, the magnitude of these differences varied considerably. While the mean differences in the vertical and anterior‐posterior dimensions were modest at 1–2 mm, the difference in the horizontal dimension (medialization) was substantially larger, averaging 5 mm. This finding suggests that the difference in bone preservation observed between the two groups was primarily driven by this reduction in medialization. Despite these differences in cup position, the mean cup non‐coverage ratio did not differ significantly between the H1 cup and the BHR cup, suggesting that the reduced bone resection achieved with H1HR was not at the expense of increased functional non‐coverage.

Previous literature strongly supports minimizing medialization as the most critical factor for bone preservation. The simulation study by Lavigne et al. demonstrated that increasing the reamer diameter from 58 mm to 60 mm resulted in a 59% increase (from 6185 mm^3^ to 9806 mm^3^) in resected volume, whereas a 3 mm increase in medialization with a 58 mm reamer produced a disproportionately larger 103% increase (from 6185 mm^3^ to 12568 mm^3^) in resected volume [30]. Furthermore, the simulation study by Raj et al. also reported that for a 56 mm cup, reducing medialization by 3 mm preserved more bone than downsizing by one size (2 mm): 6 cm^3^ (44%) versus 5 cm^3^ (36%), respectively [31]. These findings indicate that limiting medialization contributes more significantly to bone stock preservation than cup sizing alone.

The reason the H1 cup achieved this reduced medialization, despite its larger diameter, is its unique anatomical design. The H1 cup was developed based on 3D morphological analysis, and its bilaterally symmetrical design faithfully reflects the anatomical configuration of the acetabular rim, such as the symmetry in the lengths of the ischiopubic and iliopubic grooves [14]. Unlike conventional hemispherical cups, which often require substantial medialization to avoid IPI, this design allows for stable fixation with minimal medialization [24].

Therefore, H1HR achieved superior bone preservation, despite its larger cup size, through the significant reduction in medialization afforded by its anatomical design.

This study has several limitations. First, this was a retrospective simulation study using the 3D Hip Planner software. As a virtual simulation, it may not fully replicate clinical procedures or account for intraoperative factors. However, the software itself has been demonstrated to be a valid and reliable tool for preoperative planning in HRA [20]. Furthermore, reproducibility was confirmed in this study through inter‐observer validation, with high ICCs observed among different surgeons. Given the difficulty of comparing implant options in the same patient under clinical conditions, this simulation‐based approach provides valuable insights. Second, the sample size was relatively small. However, it was justified by a formal a priori power analysis which indicated that a minimum of 5 paired samples were required to detect a clinically meaningful difference with 80% power. While our study was sufficiently powered for the primary outcome, this small sample size limits the generalizability of our findings to a broader patient population and precludes any meaningful subgroup analysis. Third, this study compared H1HR with BHR only in patients with femoral head diameters ≥ 48 mm. The comparison excluded patients with head sizes between 40 mm and 46 mm, which are typically indicated for H1HR owing to its material and design advantages. This decision was based on previous reports showing inferior outcomes in patients with BHR femoral heads < 48 mm [16]. However, since the H1 cup design remains unchanged even in smaller sizes, it is likely that similar advantages in bone preservation would be observed in patients with smaller femoral heads as well. Further studies are needed to confirm this in a cohort with smaller femoral head sizes. Fourth, cup inclination angles differed between the two HRAs. This design‐specific target could influence reaming geometry and resection depth. Although we followed each manufacturer's recommended technique, future comparative analyses using identical inclination angles could help isolate the effect of implant geometry itself. Finally, in cases with advanced osteoarthritis and prominent osteophyte formation, accurate cup positioning using the present method may be challenging. However, the CT data used in this study were obtained from patients who had previously undergone HRA (Justin Cobb), reflecting the typical patient selection criteria used in clinical practice for resurfacing procedures.

In conclusion, the 35% reduction in acetabular bone resection with H1HR compared with the widely used BHR can be attributed primarily to its anatomical design, which allows for placement with minimal medialization without a significant difference in cup non‐coverage ratio. The preservation of bone offered by H1 may be particularly valuable for young, active patients likely to require future revision surgery.

## Author Contributions

**Yuki Yamamuro:** conceptualization, data curation, formal analysis, investigation, methodology, validation, visualization, writing – original draft, writing – review and editing. **Simon Harris:** methodology, software, writing – review and editing. **Rima Nasser:** methodology, writing – review and editing. **Amir Ardakani:** methodology, validation, writing – review and editing. **Justin Cobb:** conceptualization, methodology, supervision, writing – review and editing.

## Funding

The authors have nothing to report.

## Supporting information


**Table S1:** Distribution of implant sizes. *H1HR, H1 hip resurfacing*; *BHR, Birmingham hip resurfacing*.
**Table S2:** Intraclass Correlation Coefficients of the Measurements Values are Intraclass Correlation Coefficients with 95% CI.

## Data Availability

The data that support the findings of this study are available from the corresponding author upon reasonable request.
