# Supplementary material for: Acetabular Bone Preservation in H1 Ceramic Hip Resurfacing: A Comparison With A Conventional Metal‐on‐Metal Hip Resurfacing
Source: J Orthop Res. 2026 May 7;44:e70221. doi: 10.1002/jor.70221 (PMC13150472; doi:10.1002/jor.70221)
Supplement: Supplementary file 1 — Table S1: Distribution of implant sizes. H1HR, H1 hip resurfacing; BHR, Birmingham hip resurfacing. Table S2: Intraclass Correlation Coefficients of the Measurements Values are Intraclass Correlation Coefficients with 95% CI. [file JOR-44-0-s001.docx]

**SUPPLEMENTARY MATERIALS**

**Sample size calculation details**

The parameters for the a priori power analysis presented in the main text were estimated from a preliminary analysis. This analysis was conducted on the first 10 pairs of the study cohort. The mean difference in the primary outcome (volume of acetabular bone resection) between the H1 and BHR cups was found to be 8 cm³ with a standard deviation of the difference of 4 cm³. These values were used as input for the paired t-test calculation in G*Power to determine the required sample size.

| **Implant Type** | **Head Size (mm)** | **Cup Size (mm)** | **N (hips)** |
| --- | --- | --- | --- |
| H1HR | 48 | 55 | 5 |
|  | 50 | 57 | 1 |
|  | 52 | 59 | 2 |
|  | 54 | 61 | 2 |
|  | 56 | 63 | 1 |
|  | 58 | 65 | 1 |
| BHR | 48 | 54 | 5 |
|  | 50 | 56 | 2 |
|  | 52 | 58 | 2 |
|  | 54 | 60 | 2 |
|  | 56 | 62 | 1 |

**Supplementary Table SⅠ.** Distribution of implant sizes

*H1HR, H1 hip resurfacing; BHR, Birmingham hip resurfacing*

|  | | |
| --- | --- | --- |
| **Parameter** | **Intrarater** | **Interrater** |
| Head size (95% CI) | 0.98 (0.96–0.99) | 0.97 (0.92–0.99) |
| Cup size (95% CI) | 0.98 (0.96–0.99) | 0.98 (0.92–0.99) |
| Volume of acetabular bone resection (95% CI) | 0.94 (0.85–0.97) | 0.89 (0.75–0.95) |
| Horizontal distance (95% CI) | 0.99 (0.98–1.00) | 0.94 (0.78–0.98) |
| Vertical distance (95% CI) | 0.94 (0.86–0.97) | 0.89 (0.75–0.95) |
| Anterior**–**posterior distance (95% CI) | 0.96 (0.91–0.98) | 0.87 (0.71–0.95) |
| Cup non-coverage ratio (95% CI) | 0.94 (0.85–0.97) | 0.89 (0.75–0.95) |

**Supplementary Table SⅡ.** Intraclass Correlation Coefficients of the Measurements

Values are Intraclass Correlation Coefficients with 95% CI.

*CI, confidence interval*
